# Multisystem Resiliency as a Predictor of Physical and Psychological Functioning in Older Adults With Chronic Low Back Pain

**DOI:** 10.3389/fpsyg.2019.01932

**Published:** 2019-08-22

**Authors:** Emily J. Bartley, Shreela Palit, Roger B. Fillingim, Michael E. Robinson

**Affiliations:** ^1^Department of Community Dentistry and Behavioral Science, University of Florida, Gainesville, FL, United States; ^2^Pain Research and Intervention Center of Excellence, University of Florida, Gainesville, FL, United States; ^3^Department of Clinical and Health Psychology, University of Florida, Gainesville, FL, United States; ^4^Center for Pain Research and Behavioral Health, University of Florida, Gainesville, FL, United States

**Keywords:** resilience, multisystem, low back pain, aging, psychological, health, social support

## Abstract

Evidence supports the benefits of resilience among older adults with chronic pain. While numerous factors confer resilience, research has largely examined these measures in isolation, despite evidence of their synergistic effects. Conceptualizing resilience from a multisystem perspective may provide a deeper understanding of adaptive functioning in pain. Sixty adults (ages 60+ years) with chronic low back pain completed measures of physical function, pain intensity, disability, and a performance-based task assessing back-related physical functioning and movement-evoked pain (MEP). Depressive symptoms, quality of life, and general resilience were also evaluated. To examine multisystem resiliency, principal components analysis (PCA) was conducted to create composite domains for psychological (positive affect, hope, positive well-being, optimism), health (waist–hip ratio, body mass index, medical comorbidities), and social (emotional, instrumental, informational support) functioning measures, followed by cluster analysis to identify participant subgroups based upon composites. Results yielded four clusters: Cluster 1 (high levels of functioning across psychological, health, and social support domains); Cluster 2 (optimal health and low psychosocial functioning); Cluster 3 (high psychological function, moderate-to-high social support, and poorer health); and Cluster 4 (low levels of functioning across the three domains). Controlling for sociodemographic characteristics, individuals with a more resilient phenotype (Cluster 1) exhibited lower levels of disability, higher quality of life and psychological functioning, and greater functional performance when compared to those with a lower degree of personal resources (Cluster 4). No significant cluster differences emerged in self-reported pain intensity or MEP. These findings signify the presence of resiliency profiles based upon psychological, social, and health-related functioning. Further examination of the additive effects of multiple adaptive behaviors and resources may improve our understanding of resilience in the context of pain, informing novel interventions for older adults.

## Introduction

Older adults represent the fastest growing population in the United States. As such, increased attention on enhancing the health and well-being of this cohort is imperative. Among health complaints and chronic medical conditions, pain remains a significant area of concern in aging adults, with approximately 18.7 million (53%) adults ages 65 years and older (Patel et al., 2013) reporting they experience bothersome pain (Helme and Gibson, 2001). Further, chronic low back pain (cLBP) impacts 36% of this population and is the leading cause of disability in older adults (Weiner et al., 2003; Molton and Terrill, 2014). In spite of the significant burden of chronic pain in older adults, this group is often subjected to inadequate assessment and suboptimal treatment of pain (Gibson and Lussier, 2012; Molton and Terrill, 2014).

Traditionally, aging has been viewed as a period of frailty, vulnerability, and decline. However, there is considerable variability in the aging process. Indeed, the importance of considering the role of adaptive constructs in promoting successful aging (characterized by decreased disability, greater health-related functioning, and better life engagement) has been highlighted (Rowe and Kahn, 1997, 2015). Understanding factors that could delay or prevent aging-related illnesses and support successful aging would allow for the development of approaches that attenuate disability related to these health conditions. Thus, in the context of functional limitations and decreased quality of life associated with chronic pain, greater emphasis should be placed on identifying factors that ultimately inform targeted interventions for pain in older adults. These investigations should account for the multidimensional nature of pain and the myriad biopsychosocial elements that influence it.

Diminished functioning (e.g., physical disability and work-related interference) and psychosocial interference (e.g., depressed mood, anxiety, pain-related fear, and limited social support) that often accompany chronic pain play a role in disrupted quality of life in individuals with pain. To date, existing research has primarily focused on risk and vulnerability factors related to the maintenance and exacerbation of pain. For example, negative psychological factors (e.g., negative affective states) have consistently been shown to facilitate pain and disability; depression and anxiety are highly comorbid with chronic pain and can significantly impact the pain experience, leading to greater pain severity, impaired functioning, and reduced quality of life (Bair et al., 2003; Lerman et al., 2015). In fact, evidence suggests that in older adults, depression can uniquely contribute to increased risk of developing disabling back pain (Reid et al., 2003). Similarly, reciprocal relationships between symptoms of anxiety and depression and greater pain interference have been demonstrated in the aging population (Arola et al., 2010).

Additionally, negative pain beliefs (e.g., pain catastrophizing and fear-avoidance) are known to adversely influence pain-related outcomes. Consistent evidence suggests that pain catastrophizing (pain-associated rumination, magnification, and helplessness) leads to enhanced pain and greater affective disturbance (Turner and Clancy, 1986; Sullivan et al., 2001). Likewise, individuals may develop a fear of pain and movement that facilitates avoidance of certain activities following a painful injury, when they view these activities as having the potential to cause re-injury and subsequent pain (Vlaeyen and Crombez, 1999; Vlaeyen and Linton, 2000; Crombez et al., 2012). These fear-avoidance beliefs can increase pain and functional impairment, such as physical deconditioning arising from limited mobility (Rainville et al., 2011; Wertli et al., 2014). Although informative, an emphasis on pathology/vulnerability does not capture the impact of additional contributors on the pain experience, including the potentially protective role of positive, adaptive factors on chronic pain.

While aging has been regarded as a period of loss, this view has been contrasted by mounting evidence that older adults have the capacity for resilience; evidenced by high levels of reported well-being, quality of life, and self-rated successful aging, despite worsening health and substantive physical challenges (i.e., pain) (MacLeod et al., 2016). Although there are competing approaches to the conceptualization and measurement of resilience, it has largely been characterized as a trajectory of positive adaptation in response to significant risk or adversity (Ong et al., 2009). Resilience has also been delineated as a trait-like construct, consisting of personality characteristics and stable psychosocial factors that contribute to adaptive functioning; however, it is argued that this definition lacks precision as it overlooks time-varying and contextually dependent aspects of resilient responding. Further, characterization of resilience as purely dispositional fails to account for the malleability of human functioning or the consideration of how resilience can be promoted through therapeutic intervention. More recent theoretical models have conceptualized resilience as a dynamic process, characterized as an interplay between trait-based resources (e.g., personality factors) and active mechanisms (e.g., cognitive and affective states) that influence adaptive coping responses to pain. This process, in turn, promotes sustainability in meaningful and valued activities, personal growth as a result of one’s experience with chronic pain, and the capacity to recover or rebound from disruptions in physiological, emotional, or cognitive functioning (e.g., pain flare-up) (Sturgeon and Zautra, 2010, 2013).

Abundant literature has identified multiple psychological contributors to resilience. For example, optimism (Ferreira and Sherman, 2007; Goodin and Bulls, 2013; Goodin et al., 2013; Cousins et al., 2014), hope (Berg et al., 2008; Howell et al., 2015; Bartley et al., 2019b), positive affect (PA) (Zautra et al., 2005; Finan and Garland, 2015; Hassett and Finan, 2016), self-efficacy (Wright et al., 2008; Wylde et al., 2012; Brembo et al., 2017; Martinez-Calderon et al., 2018; Karasawa et al., 2019), and pain acceptance (McCracken, 1998; Kratz et al., 2007; Jensen et al., 2016) have been associated with adaptive changes across a number of pain and mental health outcomes. Perceived social support also shows benefits in individuals with chronic pain, which may be particularly relevant for older adults as social engagement provides a means of coping with pain (Molton and Terrill, 2014). In fact, perceptions of support are associated with fewer depressive symptoms (Ferreira and Sherman, 2007; Lopez-Martinez et al., 2008; Lee et al., 2016; McKillop et al., 2017), greater quality of life (Ethgen et al., 2004), lower pain intensity (Lopez-Martinez et al., 2008), and improvements in postsurgical (i.e., lower-limb amputation) functioning (Hanley et al., 2004).

Together with psychological and social functioning, numerous lifestyle and health factors also contribute to resilience. Tobacco usage is associated with a greater incidence and prevalence of pain (Goldberg et al., 2000; Shiri et al., 2010b), while multimorbidity has profound consequences on the occurrence (Schneider et al., 2007) and worsening of pain and physical functioning (Calders and Van Ginckel, 2018). Similarly, sleep has been posited as a key regulator of pain modulation, with effects on somatosensory sensitivity (Campbell et al., 2015; Schrimpf et al., 2015), pain severity (Gerhart et al., 2017), and interference (Kothari et al., 2015). Although sleep and pain are temporally related, sleep quality appears to have a more robust influence on pain symptomatology than vice versa (Finan et al., 2013; Gerhart et al., 2017), and may even serve as a risk factor for pain development and chronification (Gupta et al., 2007; Finan et al., 2013). Likewise, intervening on sleep may have salutary effects on pain, with recent evidence highlighting the influence that treatment-related sleep improvements have on pain intensity (de la Vega et al., 2019). Exercise as a therapeutic modality also confers many health benefits but can be especially potent for pain symptomatology. Increasing evidence suggests that sedentary behavior is inversely associated with functional performance (Lee et al., 2015), with greater physical activity predicting more optimal long-term outcomes in pain and disability (Pinto et al., 2014). Also, acute bouts of exercise yield analgesic effects on pain-evoked laboratory measures (Burrows et al., 2014), yet appear to be differentially influenced by physical activity behavior (e.g., sedentarism and level of physical activity) (Naugle et al., 2017; Ohlman et al., 2018). In turn, sedentary behavior may promote greater adiposity (i.e., body mass index and waist–hip ratio) which can be a risk factor for pain and functional disability (Fanuele et al., 2002; Shiri et al., 2010a; Walsh et al., 2018), presumably through a myriad of pathways such as increased joint loading, biochemical mediators, and mood disturbance (Okifuji and Hare, 2015). The association between pain and obesity is likely reciprocal, however, with chronic pain also potentiating risk for weight gain. Given that obesity is a potentially modifiable factor, some studies have highlighted the efficacy of weight loss interventions in reducing the incidence and severity of pain (Hughes et al., 2018; Dunlevy et al., 2019).

Taken together, there is a wealth of literature supporting the protective effects of psychological, social, and lifestyle factors in the experience of pain. However, much of research has examined these factors in isolation, with limited consideration of their additive contributions. Even more, while existing conceptualizations of resilience have varied widely, it has commonly been defined as a trait-based construct comprised primarily of psychological facets (Windle et al., 2011). Thus, prevailing approaches to the study of resilience may not fully capture the multidimensionality of the construct or how resilient functioning can be promoted through various systems. Extending our current conceptual models may carry important implications in terms of explicating the resources and mechanisms that promote adaptive pain outcomes. Only a modest literature has addressed the notion of multisystem resiliency. For instance, Agrigoroaei and Lachman (2011) found that a protective composite of psychosocial and behavioral factors (i.e., control beliefs, social support quality, and physical activity) predicted cognitive functioning, above and beyond the effects of sociodemographics, physical health, and cognitive activity engagement. Further, the combination of low-risk lifestyle factors (i.e., smoking, physical activity, adiposity, alcohol use, and diet) was more robustly associated with longer leukocyte telomere length (a marker of cellular aging) in women, as compared to the independent effects of each factor (Sun et al., 2012). Similarly, Johnson et al. (2019) found that a psychosocial and behavioral index of resilience [i.e., optimism, PA, negative affect (NA), active coping, perceived stress, social support, tobacco use, and waist–hip ratio] had a stronger association with telomere length in older adults with knee pain, relative to a composite comprised solely of psychological functioning measures. Overall, these findings provide compelling support for an integrative approach to studying resilience and underscore the importance of exploring these contributions in chronic pain.

The current study sought to address this gap in the literature by examining the association of multisystem resiliency with pain and psychological outcomes in a sample of older adults with cLBP. Given the dimensionality of resilience, several psychosocial resources (i.e., PA, hope, positive well-being, optimism, and social support) and health/lifestyle variables (i.e., waist–hip ratio, body mass index, physical health comorbidities, and smoking status) were considered for inclusion. These measures were selected as they represent modifiable factors with strong, empirical support for their impact on pain and health-related processes. Therefore, the primary aims were to: (1) empirically identify domains of resilience based upon psychological, social, and health-related factors and (2) using cluster analysis, explore whether resiliency phenotypes differ across measures of physical function, pain intensity, disability, and psychological functioning. It was hypothesized that: (1) homogenous subgroups would emerge from patterns of psychological, health, and social resiliency and (2) individuals with more resilient phenotypes (i.e., higher in protective resources) would exhibit higher physical function, lower self-reported pain and disability, and greater psychological functioning.

## Materials and Methods

### Participants and Procedures

This was a cross-sectional study based on a secondary data analysis from the Adaptability and Resilience in Aging Adults (ARIAA) study, a project evaluating the effects of resilience mechanisms on pain modulatory capacity among individuals with cLBP. Sample size estimations were based upon previous pilot data (Bartley et al., 2019b) establishing that 60 participants would provide power of 0.80 at 0.05 (two-tailed) for detecting moderate to large effect sizes between measures of resilience and pain.

Older adults (ages 60+ years) with cLBP (*N* = 69) were recruited from the community via posted fliers, media announcements, and word-of-mouth referral. All participants provided verbal and written informed consent. Participants were included if they reported at least mild LBP (≥2/10) occurring on at minimum half of the days during the preceding 3 months. Enrollment in the study was not limited to LBP (due to the presence of medical comorbidities in this population) as long as LBP was an individual’s primary pain condition. Exclusion criteria were as follows: recent vertebral fracture; back surgery within the past 6 months; diagnosis of cauda equina syndrome; uncontrolled hypertension (≥150/90); severe cardiovascular disease (e.g., recent heart attack); neurological disease associated with somatosensory abnormalities (e.g., neuropathy, seizures, and Parkinson’s disease); current major medical illness (e.g., metastatic or visceral disease); chronic opioid use; and systemic inflammatory disease (e.g., spondylarthropathies such as ankylosing spondylitis and systemic lupus erythematosus). Participants were provided up to $100 compensation upon completion of the study.

The University of Florida Institutional Review Board approved all study procedures. Initially, participants were evaluated for study inclusion and exclusion through a brief telephone screen. The following sociodemographic and health data were obtained as part of the screening: self-reported sex, age, and a brief health history including the presence of major medical illnesses, recent back-related injuries or surgeries, and LBP symptoms. If eligible, participants attended two, 2–3.5-h appointments scheduled approximately 1 week apart. During Session 1, eligibility criteria were verified through a self-reported demographic and medical history assessment, and participants completed anthropometric tests, psychosocial questionnaires, and functional performance measures. During the time in between Sessions 1 and 2, participants completed several questionnaires at home. Sensory pain testing was conducted during Session 2 (data not reported), and additional psychosocial questionnaires were also completed at that visit.

### Measures

#### Predictors of Multisystem Resilience

##### Positive and negative affect schedule

The 20-item Positive and Negative Affect Schedule (PANAS) was used to examine PA and NA (Watson et al., 1988). Respondents were presented with 10 positively valenced and 10 negatively valenced terms that are rated on a five-point scale ranging from 1 (very slightly or not at all) to 5 (extremely) resulting in scale scores for PA and NA, with higher scores indicating increased positive and NA, respectively (only PA scores were included in the current analysis). Reliability tests indicated high internal consistency of items on the PA scale (α = 0.90).

##### Adult dispositional hope scale

The Adult Dispositional Hope Scale (ADHS) is a 12-item questionnaire that includes eight statements measuring two aspects of hope: pathways (e.g., “There are lots of ways around a problem.”) and agency (e.g., “I energetically pursue my goals.”), as well as four “filler” statements that are not included in scoring (Snyder et al., 1991). Items are rated on a scale ranging from 1 (definitely false) to 8 (definitely true) and respondents select the number that best describes them for each statement. Higher scores indicate greater trait levels of hope. Reliability analyses from the current investigation revealed Cronbach’s α for the ADHS = 0.92, indicating high internal consistency for this measure.

##### PROMIS positive affect and well-being scale

The Patient-Reported Outcomes Measurement Information System (PROMIS) PA and Well-Being Scale was used to measure PA and overall sense of satisfaction with life (Salsman et al., 2013). This scale consists of 23 items rated on a 1 (never) to 5 (always) scale to indicate how often respondents experienced positive emotion and/or purpose/meaning in life (e.g., “[Lately], I had a sense of balance in my life.”). Higher scores reflect greater PA and well-being (Cronbach’s α = 0.97).

##### Life-orientation test-revised

Dispositional optimism was evaluated using the Life-Orientation Test-Revised (LOT-R), which consists of 10 items (including four unscored items and three reverse-scored items). Participants were asked to use a 5-point scale ranging from 0 (strongly disagree) to 4 (strongly agree) and rate the degree to which they agreed with the presented statements (e.g., “In uncertain times, I usually expect the best.”) (Herzberg et al., 2006). Higher LOT-R scores indicate greater optimism. This measure demonstrated adequate reliability in the sample (α = 0.73).

##### PROMIS support (emotional, instrumental, informational)

To measure social functioning, the short forms of the PROMIS emotional (eight items; e.g., “I have someone who makes me feel appreciated.”), instrumental (four items; e.g., “Do you have someone to take you to the doctor if you need it?”), and informational (four items; e.g., “I have someone to turn to for suggestions about how to deal with a problem.”) support scales were administered (Hahn et al., 2014). Items are rated on a 1 (Never) to 5 (Always) scale for all three domains, with higher scores indicating greater social support. All three scales were found to have high internal consistency and were also highly reliable with each other: emotional (α = 0.97), instrumental (α = 0.96), informational (α = 0.96), all support measures combined (α = 0.97).

##### Anthropometric tests: body composition

During Session 1, participants’ waist (5 cm above the navel) and hip circumferences (widest part of the hips) were calculated (in cm) using a measuring tape, with waist–hip ratio determined by dividing the waist circumference by the hip circumference. Body weight was measured to the nearest 0.1 kg using a digital scale (Healthometer) and height was assessed to the nearest centimeter using a wall stadiometer. Calculation of BMI was determined by weight in kilograms divided by height in meters squared.

##### Health comorbidities

To determine the presence of physical health comorbidities, participants completed a health status questionnaire whereby they were asked to place an “X” next to any current medical conditions (i.e., high blood pressure, heart disease, diabetes, asthma/breathing problems, kidney/renal disease, thyroid problem, neurological disorder, or other self-reported health conditions). Medical diagnoses were placed into ICD-10 diagnostic categories for reporting purposes.

##### Smoking status

Current cigarette smoking status was assessed using the following question: “How would you describe your cigarette smoking?” Possible responses included: “never smoked,” “used to smoke but have now quit,” and “current smoker,” and individuals were categorized as either current smokers (yes) or non-smokers (no).

#### Study Outcomes

##### Back performance scale

Functional performance and movement-evoked pain (MEP) were measured using the Back Performance Scale (BPS). The BPS consists of a series of tasks (i.e., Sock Test, Pick-up Test, Roll-up Test, Fingertip-to-Floor Test, and Lift Test) that are designed to measure functional capacity during completion of mobility-oriented activities that have been deemed to be particularly difficult for individuals with back pain (Magnussen et al., 2004; Strand, 2017). An evaluator assesses the degree to which these tasks are completed. Physical functioning scores range from 0 to 3 for each test (total scale score = 0–15), with increasing scores indicating greater difficulty with task performance. MEP was measured by asking participants to rate their current LBP from 0 (no pain) to 100 (most intense pain imaginable) immediately after completion of each of the five tasks on the BPS. MEP was determined from an average of the five pain ratings. Internal consistency was good for this measure (α = 0.83).

##### PROMIS physical function

To evaluate the general physical functioning, the short form of the PROMIS Physical Function measure was administered (Rose et al., 2008, 2014). This scale includes four questions (e.g., “Are you able to do chores such as vacuuming or yard work?”) to examine the difficulty with which an individual is able to complete certain functional tasks. Ratings are made from 5 (without any difficulty) to 1 (unable to do) and lower scores indicate greater difficulty with task performance. This measure demonstrated high reliability among the sample (α = 0.85).

##### PROMIS pain intensity

The three-item PROMIS Pain Intensity short form measure was used to evaluate pain intensity over the past week (Cella et al., 2010). This scale asks respondents to report their average and worst pain during the past 7 days, as well as pain at the time of questionnaire completion by providing a 1 (no pain) to 5 (very severe) pain rating. The PROMIS Pain Intensity scale demonstrated good reliability (α = 0.81).

##### Roland-morris disability questionnaire

The Roland-Morris Disability Questionnaire (RMDQ) is a self-report measure that assesses health status and disability related to LBP (Roland and Morris, 1983). The RMDQ is comprised of 24 statements such as “I stay at home most of the time because of my back” and “I only walk short distances because of my back.” Respondents are instructed to indicate which of the statements describe their current experience. The number of endorsed items is summed to obtain a total score (more items endorsed = greater disability). Internal consistency was high for this measure (α = 0.87).

##### PROMIS depression scale

The eight-item short form of the PROMIS Depression Scale was used to assess depressive symptoms (e.g., “I felt worthless.”) (Pilkonis et al., 2011). Respondents rate the frequency of their experience of each symptom in the past 7 days from 1 (never) to 5 (always), with higher scores indicating a greater presence of depressive symptoms. The PROMIS Depression Scale demonstrated high reliability (α = 0.93).

##### Brief resilience scale

Trait resilience was examined using the Brief Resilience Scale (BRS), which is a six-item measure examining the ability to bounce back and recover from stressful events and challenges (e.g., “I tend to bounce back quickly after hard times.”) (Smith et al., 2008). Responses are provided using a five-point scale (1 = strongly disagree, 5 = strongly agree), with total scores ranging from 6 to 30. Higher scores on the BRS indicate greater resilience (Cronbach’s α = 0.84).

##### World health organization quality of life-brief

The World Health Organization Quality of Life-Brief (WHOQOL-BREF) is a 26-item questionnaire designed to measure quality of life across four domains over the past 2 weeks: physical health, psychological health, social relationships, and environment (Skevington et al., 2004). The first item of the WHOQOL-BREF (i.e., “How would you rate your quality of life?”) was used to examine overall quality of life. This item is rated from 1 (very poor) to 5 (very good).

### Statistical Analysis

All analyses were conducted using SPSS 24 and significance level was set at *p* ≤ 0.05 (two-tailed). Means, standard deviations, and counts for demographic characteristics were calculated using descriptive statistics. Zero-order correlations were conducted between sociodemographic characteristics and outcome variables (i.e., physical function, MEP, pain intensity, back-related disability, depressive symptoms, general resilience, and quality of life). Demographic variables that were significantly related to outcome variables were controlled for in cluster analyses. The following 11 variables were entered into a PCA to characterize the dimensionality of each resilience measure: PA, dispositional hope, positive well-being, optimism, waist–hip ratio, body mass index, physical health comorbidities, smoking status, emotional support, instrumental support, and informational support. PCA with oblique rotation was used to allow for correlation between factors, with the recommendation that at least three items load on a factor and a difference of ≥0.20 was present between cross-loadings (Howard, 2016). Components with eigenvalues >1 were selected for further analysis and the scree plot was inspected to confirm the number of factors to be retained. Hierarchical cluster analysis employing Ward’s clustering method with squared Euclidean distances as the similarity measures was conducted to identify subgroups of individuals that differed across empirically derived resilience domains. Agglomeration coefficients were examined to identify the cluster solution that best represented the data, with the optimal number being chosen based upon the point at which the percentage change was the largest between the clusters (Milligan and Cooper, 1985). Chi-square analysis for categorical variables or analysis of variance (ANOVA) for continuous variables was employed to examine cluster group differences across demographic composition. Differences across physical function, pain, and psychological outcomes were assessed using multivariate ANOVA’s, controlling for the effects of relevant sociodemographic characteristics. Significant findings on multivariate analyses were followed by Sidak-corrected *post hoc* comparisons. To obtain effect size estimates associated with *F-*tests, partial eta-squared ($\eta_{\text{p}}^{2}$) was calculated (small = 0.01, medium = 0.06, and large = 0.14).

## Results

### Participant Characteristics

Demographic characteristics (means and *SD*s) are reported in Table 1. Participants were mostly female (57%), White/Caucasian (70%), had a college degree (50%), were married or partnered (52%), and were not employed (85%). Average age was 68 years (range: 60–93 years), duration of back pain was 16.4 years (range: 1–56 years), and participants reported back pain of moderate intensity during the initial session (*M* = 5.5, range = 2–10). Two of the 69 participants discontinued after the first session due to time constraints, and 7 participants who were initially eligible were excluded during their first appointment (*n* = 1 use of exclusion medications, *n* = 3 exclusionary medical condition, *n* = 3 not meeting pain duration criteria), thus leaving 60 participants. Based on ICD-10 classifications (World Health Organization, 2016), the following medical comorbidities/diseases were reported: circulatory and respiratory (*n* = 27, 45.0%), metabolic and endocrine (*n* = 14, 23.3%), genitourinary and renal (*n* = 4, 6.7%), digestive (*n* = 3, 5.0%), skin/subcutaneous tissue (*n* = 3, 5.0%), eye (*n* = 3, 5.0%), musculoskeletal (*n* = 1, 1.6%), nervous system (*n* = 1, 1.6%), infectious disease (*n* = 3, 5.0%), and sleep disorders (*n* = 2, 3.3%). Current smoking was reported among 16.7% (*n* = 10) of the sample.

**TABLE 1:** Demographic and clinical characteristics.

			**Cluster 1**		**Cluster 2**		**Cluster 3**		**Cluster 4**	
					**High health**		**High PsySoc**			
	**Total sample**		**High resilience**		**low PsySoc**		**low health**		**Low resilience**	
	**(*n* = 60)**		**(*n* = 25)**		**(*n* = 13)**		**(*n* = 15)**		**(*n* = 7)**	
**Characteristic**	**M or N**	**SD or %**	**M or N**	**SD or %**	**M or N**	**SD or %**	**M or N**	**SD or %**	**M or N**	**SD or %**
Age (years)	68.1	7.0	69.1	5.8	65.4	5.1	70.0	10.2	65.6	4.7
**Sex**										
Male	26	43.3	11	44.0	3	23.1	9	60.0	3	42.9
Female	34	56.7	14	56.0	10	76.9	6	40.0	4	57.1
**Race**										
White/Caucasian	42	70.0	19	76.0	8	61.5	10	66.7	5	71.4
Black/African American	12	20.0	4	16.0	3	23.1	4	26.7	1	14.3
Other	6	10.0	2	8.0	2	15.4	1	6.7	1	14.3
**Education**										
≤HS diploma	13	21.7	3	12.0	4	30.8	5	33.3	1	14.3
Some college/tech degree	17	28.3	7	28.0	3	23.1	3	20.0	4	57.1
Associates/bachelors	18	30.0	11	44.0	4	30.8	2	13.3	1	14.3
Graduate/professional	12	20.0	4	16.0	2	15.4	5	33.3	1	14.3
**Marital status**										
Married/partnered	31	51.7	21	84.0	2	15.4	6	40.0	2	28.6
Not married/partnered	29	48.3	4	16.0	11	84.6	9	60.0	5	71.4
**Employment**										
Employed	9	15.0	4	16.0	2	15.4	2	13.3	1	14.3
Not employed	51	85.0	21	84.0	11	84.6	13	86.7	6	85.7
**Income^∗^**										
≤$20,000	21	35.0	5	20.8	5	38.5	7	50.0	4	66.7
$20,000–39,999	10	16.7	3	12.5	5	38.5	1	7.1	1	16.7
$40,000–59,999	11	18.3	7	29.2	1	7.7	3	21.4	0	0.0
$60,000–99,999	8	13.3	6	25.0	2	15.4	0	0.0	0	0.0
≥$100,000	7	11.7	3	12.5	0	0.0	3	21.4	1	16.7
Back pain duration (years)	16.4	14.2	20.8	16.1	15.8	12.9	9.9	10.8	16.0	12.7

*^∗^Some data not reported. HS, high school.*

### Zero-Order Correlations

To identify potential study covariates, zero-order correlations were analyzed across sociodemographic variables and study outcomes (Table 2). In general, age, sex, race, education, marital status, employment, income, and back pain duration were associated with physical function, pain, and psychological outcomes (all *p*s < 0.04). Hence, analyses assessing cluster group differences across study outcomes included these sociodemographic variables as statistical covariates.

**TABLE 2:** Zero-order correlations across sociodemographic characteristics and study outcomes.

	**BPS function**	**BPS pain**	**PROMIS function**	**PROMIS pain**	**RMDQ disability**	**PROMIS depression**	**BRS resilience**	**WHOQOL QOL**
Age	0.05	–0.18	0.01	–0.23	–0.11	−0.30^∗^	0.29^∗^	0.27^∗^
Sex	0.18	0.29^∗^	–0.20	0.24	0.23	–0.09	0.09	–0.05
Race	0.16	0.28^∗^	−0.32^∗^	0.42^∗∗^	0.36^∗∗^	0.02	–0.16	0.03
Education	–0.12	−0.25^∗^	0.20	–0.35^∗∗^	–0.38^∗∗^	–0.03	0.23	0.05
Marital status	–0.13	0.03	–0.25	0.25^∗^	0.21	0.30^∗^	–0.37^∗∗^	–0.20
Employment	0.14	0.26^∗^	–0.22	0.26^∗^	0.22	0.05	–0.12	–0.01
Income	0.01	–0.36^∗∗^	0.33^∗^	–0.55^∗∗^	–0.43^∗∗^	−0.32^∗^	0.45^∗∗^	0.45^∗∗^
Pain duration	–0.00	−0.26^∗^	0.28^∗^	–0.13	–0.35^∗∗^	–0.14	0.07	0.16

*p ≤ 0.05, **p ≤ 0.01. Sex coded: 0 = male, 1 = female; Race coded: 0 = white, 1 = black/other; Marital Status coded: 0 = married/partnered, 1 = not married/partnered; Employment coded: 0 = employed, 1 = not employed. BPS, Back Performance Scale; PROMIS, Patient-Reported Outcomes Measurement Information System; RMDQ, Roland-Morris Disability Questionnaire; BRS, Brief Resilience Scale; WHOQOL, World Health Organization Quality of Life questionnaire.

### Principal Components Analysis

A PCA was conducted with all 11 items using oblique rotation (direct oblimin), resulting in a four-factor solution. However, on the basis of our item selection criteria (i.e., more than or equal to three items load on a factor), this solution was eliminated as it returned one component containing smoking status. This variable was therefore removed from the model. The resulting analysis revealed the presence of a three-factor solution with eigenvalues over Kaiser’s criterion of 1, accounting for 72.4% of the variance in scores. The Kaiser–Meyer–Olkin measure verified the sampling adequacy for the analysis (Kaiser–Meyer–Olkin = 0.78; Bartlett’s test of sphericity = <0.001) and all KMO values were above the acceptable limit of 0.50 (Field, 2013). Inspection of the scree plot confirmed inflexions that would justify retaining three factors. Table 3 reports the factor loadings after rotation, with Component 1 representing positive, psychological factors (factor loadings 0.67–0.91), Component 2 denoting health-related functioning (factor loadings 0.60–0.78), and Component 3 reflecting social support (factor loadings 0.77–0.86). The factor loadings from each domain were used in subsequent cluster analysis.

**TABLE 3:** Principal components analysis loadings across resilience domains.

**Measures**	**Factor 1**	**Factor 2**	**Factor 3**
Positive affect	**0.91**	0.01	–0.03
Dispositional hope	**0.85**	0.03	0.08
Positive well-being	**0.69**	0.02	0.42
Optimism	**0.67**	–0.19	0.14
Waist–hip ratio	–0.20	**0.78**	0.30
Body mass index	–0.04	**0.77**	–0.04
Health comorbidities	0.34	**0.60**	–0.36
Emotional support	–0.00	0.09	**0.86**
Instrumental support	0.26	–0.05	**0.80**
Informational support	0.33	–0.03	**0.77**
Eigenvalue	4.36	1.59	1.29
% Variance	43.58	15.91	12.88
% Cumulative variance	–	59.49	72.37

*Bolded values were retained in each component.*

### Cluster Analysis Across Resilience Domains

**FIGURE 1:**
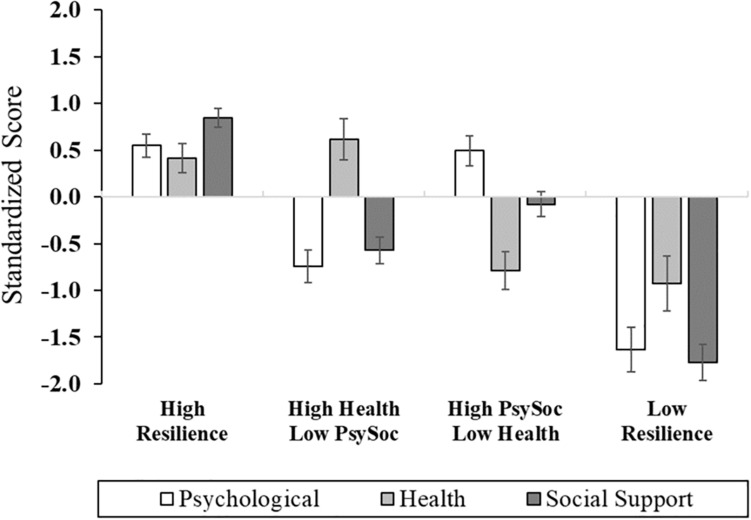
Cluster group differences across multisystem resilience domains comprised of psychological, health, and social functioning.

The three composite domains were subjected to Cluster Analysis to identify empirically derived classifications based upon profiles of psychological, health, and social resiliency (Figure 1 and Supplementary Table 1). For ease of interpretation, the health domain was reverse scored, such that lower scores reflected higher waist–hip ratio, body mass index, and health comorbidities. Four clusters were revealed and characterized by the following: (1) Cluster 1: High Resilience group (*n* = 25, 41.7%): high levels of psychological, health, and social support functioning; (2) Cluster 2: High Health/Low Psychosocial group (*n* = 13, 21.7%): optimal health-related functioning and low levels of psychosocial function; (3) Cluster 3: High Psychosocial/Low Health group (*n* = 15, 25.0%): poor health functioning, high psychological functioning, and moderate-to-high social support; and (4) Cluster 4: Low Resilience group (*n* = 7, 11.7%): low levels of functioning across psychological, social, and health-related factors. There were no sociodemographic differences across cluster groups, with the exception of the High Resilience group (Cluster 1) having the highest proportion of participants who were married or partnered (Table 1); thereby, consistent with previous research (Mun et al., 2019).

### Psychosocial Profiles Across Cluster Group

**FIGURE 2:**
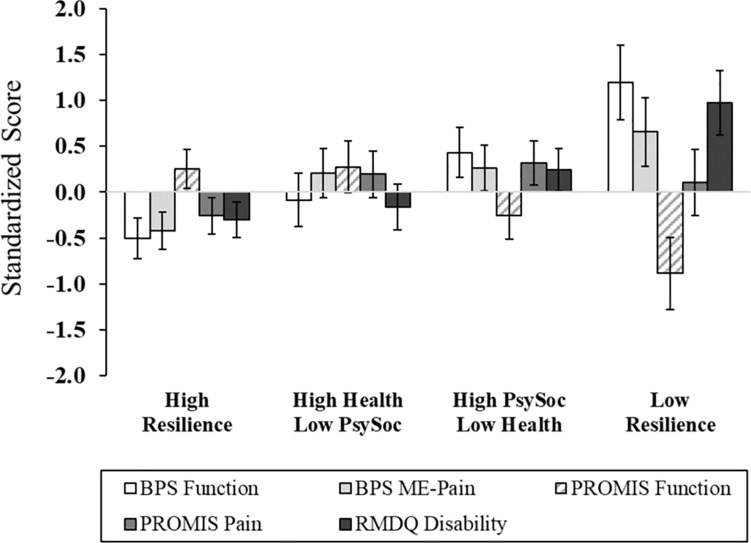
Pain and physical functioning outcomes across multisystem resilience profiles. Relative to Cluster 4 (Low Resilience group), individuals with a greater degree of protective resources had higher functional performance and self-reported physical function, as well as lower disability. There were no group differences in movement-evoked pain or pain intensity. Higher scores on PROMIS function, better physical functioning; BPS, Back Performance Scale; ME, Movement-Evoked; PROMIS, Patient-Reported Outcomes Measurement Information System; RMDQ, Roland-Morris Disability Questionnaire.

**FIGURE 3:**
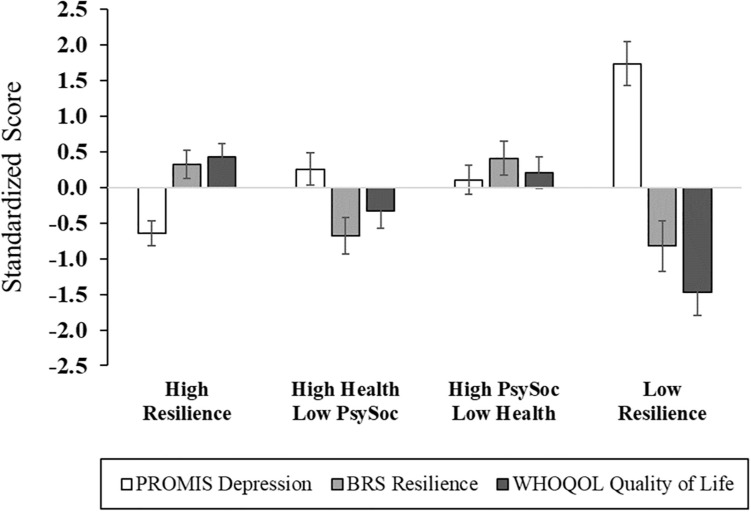
Psychological functioning across multisystem resilience profiles. Compared to Cluster 4 (Low Resilience group), individuals with more resilient phenotypes exhibited lower depressive symptoms, and higher general resilience and quality of life. PROMIS, Patient-Reported Outcomes Measurement Information System; BRS, Brief Resilience Scale; WHOQOL, World Health Organization Quality of Life questionnaire.

After adjusting for age, sex, race, education, marital status, employment, income, and back pain duration, significant differences across cluster membership emerged in functional performance, physical function, back-related functional disability, depression, general resilience, and quality of life (Figures 2, 3 and Supplementary Table 2). In particular, functional performance and functional disability due to LBP were poorest among the Low Resilience group, relative to individuals in the High Resilience and High Health/Low Psychosocial cluster groups (*p*s < 0.05). *Post hoc* comparisons were non-significant across cluster groups for self-reported physical function, although the difference between the High Health/Low Psychosocial and Low Resilience groups approached significance with a large effect size ($\eta_{\text{p}}^{2}$ = 0.22). For psychological outcomes, individuals in the Low Resilience group reported the highest levels of depression (*p*s ≤ 0.001) and lowest quality of life (*p*s ≤ 0.04), relative to all other groups. Depression was also lower in the High Resilience group (*p* = 0.05), when compared to individuals with low psychosocial resources (Cluster 2). The High Resilience (*p* = 0.04) and High Psychosocial/Low Health (*p* = 0.02) clusters had greater general resilience than the High Health/Low Psychosocial group. In addition, while the Low Resilience group reported statistically lower levels of general resilience relative to the High Psychosocial/Low Health group (*p* = 0.04), these effects only approached significance (*p* = 0.07) when compared with the High Resilience group. No differences in MEP (*p* = 0.08) or self-reported pain intensity (*p* = 0.33) were detected across cluster groups.

## Discussion

Although risk factors have been extensively studied in respect to pain, there is a burgeoning literature supporting the role of resilience mechanisms in promoting adaptive pain outcomes. Mounting evidence signifies that a multitude of psychological, social, and physical/biological factors confer resilience; however, much of the extant literature has focused on psychological resources. Moreover, resilience factors have predominantly been examined in isolation, thus overlooking their potentially synergistic and additive effects. Due to the exponential growth of older adults and global burden of chronic pain in this population, explicating the mechanisms that protect against pain and disability is of critical importance. While a modest literature has considered the cumulative effects of a broad range of personal resources (Agrigoroaei and Lachman, 2011; Sun et al., 2012; Johnson et al., 2019), we have extended previous research by capturing the multifaceted nature of resilience and exploring its influence on pain outcomes among older adults with cLBP.

Aligning with study hypotheses, we found evidence of phenotypic patterns of resilience based upon psychological, social, and health-related functioning. In particular, individuals with a higher array of protective factors exhibited more optimal outcomes in physical function, disability, and psychological processes (despite similar levels of pain), suggesting potentially important benefits of multiple adaptive resources. Overall, these findings signify that individuals with a more resilient phenotype may have a greater sense of coherence that allows them to mobilize resources to successfully navigate the ongoing challenges associated with pain. This would align with Antonovsky’s salutogenic model of health (Antonovsky, 1996) which highlights the importance of coping strengths in fostering one’s capacity for optimal health and well-being.

While a number of studies have classified patients according to negative psychological and lifestyle variables (Rabey et al., 2016; Almeida et al., 2018), limited research has stratified subgroups according to sources of resilience. In fibromyalgia, Mun et al. (2019) found that individuals with a higher degree of personal resources (i.e., pain acceptance, resilience, social support, sleep quality) exhibited lower levels of morning pain and depressive symptoms, as well as afternoon pain interference (although effects varied according to level of depression). Similar findings were also observed among patients with chronic neurological/neuromuscular disease, as those with a more resilient profile reported lower interference from pain (Mun et al., 2019). Furthermore, our findings echo a previous study in knee osteoarthritis (Cruz-Almeida et al., 2013), whereby a subgroup characterized by high optimism and low NA exhibited the lowest degree of pain, disability, and somatosensory sensitivity.

Results also suggest that health and psychosocial factors are differentially expressed across older adults with LBP. In particular, when compared to individuals with low resilience (Cluster 4), those with a higher degree of protective resources exhibited lower depression and higher quality of life. However, the findings for general resilience were more robust among individuals with higher social support and positive, psychological function (Cluster 3), thus underscoring the protective nature of psychosocial resources in coping with stress and adversity. Likewise, more favorable outcomes in functional performance and disability were not only observed among individuals with higher overall resilience, but also among those with more adaptive health-related function. This is not entirely surprising as higher disease burden and adiposity may facilitate decrements in functional capacity through mechanisms linked to frailty, psychological comorbidities, physiological dysregulation, increased joint loading, cardiopulmonary reserve, and activity restriction, among others (Fried et al., 2004; Kalyani et al., 2010; Calders and Van Ginckel, 2018). What is more, the influence of these health factors is likely not independent; rather, their effects are interactive and systemic, impacting multiple homeostatic processes to potentiate downstream effects on disability and function (Chapman et al., 2008).

Importantly, our findings have important clinical value as the protective resources we examined are modifiable. For instance, a greater emphasis on enhancing social support and positive, psychological processes may be particularly advantageous for improving adaptive coping, and to some extent, attenuating depressive symptoms in Cluster 2 individuals. Interventions with empirical support for their efficacy (e.g., cognitive-behavioral therapy and spouse-assisted training) (Edwards et al., 2016) are likely to derive some benefit; however, therapies focusing on harnessing resilience through positive, psychological resources [e.g., positive activity interventions (PAIs)] have also shown promise in chronic pain populations (Hausmann et al., 2014, 2017; Muller et al., 2016; Peters et al., 2017). For individuals with poorer health-related functioning (Cluster 3), minimizing the severity of multimorbidity and reducing weight burden through diet and exercise promotion may mitigate functional decline and disability. Ultimately, the development of strategies for the prevention of obesity and medical comorbidities is a critical directive. Results also suggest that individuals with a low resilient profile would likely benefit from a multimodal approach that optimizes both psychosocial and health-related resources. In particular, combining psychotherapy with lifestyle modification may yield protective benefits in physical and emotional functioning. For individuals in Cluster 1 who appear to be adapting well despite the presence of cLBP, these treatments may be less justified. Surprisingly, while indices of pain intensity (i.e., PROMIS pain intensity, MEP) were lower among individuals with a greater degree of resources (Cluster 1), these effects failed to reach significance across cluster groups. Although it is conceivable that other unmeasured resources may have a more robust influence on pain severity, it is also possible our study was underpowered to detect pain-specific effects. On the basis of the effect sizes observed ($\eta_{\text{p}}^{2}$ = 0.07 to 0.14), a power analysis revealed that a sample size of 72 to 150 participants would be adequate to detect significant effects in MEP and self-reported pain intensity, respectively. Thus, consideration of these findings in a larger sample is warranted.

Multiple resources shape the expression and development of resilience in chronic pain. While the measurement of resilience is inherently complex (Southwick et al., 2014) due to varying definitions and multiple methods by which to assess this construct, our current models lack precision and fail to account for the multifaceted nature of resilience. Indeed, recent theoretical literature (Liu et al., 2017) posits that resilience should be conceptualized from various levels of analysis that includes intraindividual (e.g., physiological, health behaviors), interpersonal (e.g., personality correlates, coping appraisals), and socio-ecological factors (e.g., socioeconomic status, group membership). While exploring the independent determinants that buffer against negative pain sequelae has clinical utility, recognition of resilience from a multidimensional perspective will likely provide a greater understanding of adaptive capacity. Expanding our current models of resilience and considering new approaches, both theoretically and statistically, in how resilience is conceptualized and assessed in the context of pain will be an important future direction.

### Strengths and Limitations

Several strengths of our study merit acknowledgment. To our knowledge, the current investigation is the first to examine resilience from a multidimensional perspective in older adults with cLBP. Participants were phenotyped according to several adaptive resources, offering a novel opportunity to explore how pain, disability, and psychological functioning differ across resiliency profiles. We used an empirical approach to characterize our resilience indices, which provides a statistical, data-driven, and robust method for classification of subgroups. A number of valid and reliable measures were also utilized across the assessment of psychological, social, and health-related functioning. Further, despite the small sample (*N* = 60), large effect sizes (ranging from $\eta_{\text{p}}^{2}$ = 0.17 to 0.45) were observed for pain and psychological outcomes.

In spite of these strengths, a few limitations are worth noting in the interpretation of results. First, given the nature of cluster analysis our findings should be considered exploratory. In addition, our relatively modest sample may have influenced our classification of individuals using cluster analysis, with few individuals categorized into particular profiles (e.g., only seven participants comprised the “Low Resilience” cluster). This may have impacted the external validity of the study, thereby compromising generalizability. Future studies with larger sample sizes are warranted to confirm these findings. In light of these limitations, results should be interpreted cautiously. Although we employed a robust, empirical approach to devise our resilience domains, smoking status was eliminated from the analyses due to its retention as an independent factor. Despite smoking being a relevant lifestyle behavior with tremendous health consequences, we have confidence that the omission of this variable did not alter our findings, as it only contributed a small amount of additional variance (4%) to our model. Furthermore, analyses revealed that smoking status did not differ across cluster groups (*p* = 0.18). Nevertheless, there are strengths and challenges to various statistical methods (e.g., factor analysis, *z*-scores, and median split approach) and future studies should consider the comparison of these approaches, as well as their clinical relevance. Of note, some cross-loadings (>0.30) were observed across psychological, health, and social support factors. Although this may be a limitation, this phenomenon is also anticipated given the natural correlation among these constructs (e.g., health-related constructs correlate with many other variables). Also, medical comorbidities were determined via participant self-report, which may not provide a complete representation of individuals’ health histories. Medical records should be obtained for verification of health history in future endeavors. Related, because we excluded individuals experiencing major medical conditions, these results may not generalize to those who experience more severe health comorbidities.

Although multiple psychosocial and health constructs were used to derive subgroups, this did not reflect an exhaustive list of protective resources. Indeed, the small sample limited our ability to examine several factors, including relevant physiological/biological markers (e.g., inflammatory cytokines) (Khan et al., 2017), or demographic characteristics such as sex and race/ethnicity. Moreover, there is a need to replicate these findings in a more diverse sample, especially given growing evidence of the impact of race/ethnicity on resilience and pain-related outcomes (Bartley et al., 2019a). Inclusion of these and other diversity variables will be important considerations for future studies. Employing additional measures of health/lifestyle factors (e.g., physical activity, sleep, diet, alcohol, and drug consumption), as well as other positive psychological (e.g., self-efficacy and sense of coherence) and social support-related indices (e.g., quantity versus quality of support) will be key to improving our understanding of resilience. Likewise, given the importance of external determinants of resilience, several other contextual and social/environmental factors, including socioeconomic status and access to healthcare, are critical areas to examine (Liu et al., 2017). And finally, it may be beneficial to use a dual-focus approach to closely examine both risk and resilience factors and how this interplay influences multisystem resiliency in respect to pain (perhaps even considering the “degree” of negative factors, such as the impact of lower NA, less catastrophic thinking, etc.). For example, personal resources (such as optimism or PA) may broaden an individual’s coping repertoire by facilitating engagement in adaptive behaviors that mitigate the narrowing effects of pain catastrophizing (Fredrickson, 2001). This would align with predominant risk-resilience models that highlight the consideration of both vulnerability and protective mechanisms in understanding individual adaptation to pain (Sturgeon and Zautra, 2013; Goubert and Trompetter, 2017).

## Conclusion

In sum, our findings support the contribution of protective factors in the context of pain and suggest that examining resilience from a multisystem perspective may have significant clinical utility. Importantly, homogenous subgroups emerged from psychological, social, and health-related processes, with lower disability, better functional performance, and higher psychological functioning observed among individuals with a more resilient phenotype. Consideration of the multiple resources that harness resilience, including their additive effects, may improve our understanding of adaptive function among older adults with chronic pain and ultimately facilitate the development of more targeted clinical care.

## Data Availability

The datasets generated for this study are available on request to the corresponding author.

## Ethics Statement

The studies involving human participants were reviewed and approved by the University of Florida Institutional Review Board. The patients/participants provided their written informed consent to participate in this study.

## Author Contributions

EB and SP contributed to the conception and design of the study, and wrote the first draft of the manuscript. EB performed the statistical analysis. All authors reviewed the text critically and approved the submitted version.

## Conflict of Interest Statement

The authors declare that the research was conducted in the absence of any commercial or financial relationships that could be construed as a potential conflict of interest.
