# Corrigendum: Multisystem Resiliency as a Predictor of Physical and Psychological Functioning in Older Adults With Chronic Low Back Pain

**DOI:** 10.3389/fpsyg.2020.595827

**Published:** 2020-10-12

**Authors:** Emily J. Bartley, Shreela Palit, Roger B. Fillingim, Michael E. Robinson

**Affiliations:** ^1^Department of Community Dentistry and Behavioral Science, University of Florida, Gainesville, FL, United States; ^2^Pain Research and Intervention Center of Excellence, University of Florida, Gainesville, FL, United States; ^3^Department of Clinical and Health Psychology, University of Florida, Gainesville, FL, United States; ^4^Center for Pain Research and Behavioral Health, University of Florida, Gainesville, FL, United States

**Keywords:** resilience, multisystem, low back pain, aging, psychological, health, social support

In the original article, there was a mistake in Table 2 as published. Data for the last three columns (PROMIS depression, BRS resilience, WHOQOL quality of life) were inadvertently interchanged. The corrected Table 2 appears below.

**Table 2:** Zero-order correlations across sociodemographic characteristics and study outcomes.

	**BPS function**	**BPS pain**	**PROMIS function**	**PROMIS pain**	**RMDQ disability**	**PROMIS depression**	**BRS resilience**	**WHOQOL QOL**
Age	0.05	−0.18	0.01	−0.23	−0.11	−0.30^*^	0.29^*^	0.27^*^
Sex	0.18	0.29^*^	−0.20	0.24	0.23	−0.09	0.09	−0.05
Race	0.16	0.28^*^	−0.32^*^	0.42^**^	0.36^**^	0.02	−0.16	0.03
Education	−0.12	−0.25^*^	0.20	−0.35^**^	−0.38^**^	−0.03	0.23	0.05
Marital status	−0.13	0.03	−0.25	0.25^*^	0.21	0.30^*^	−0.37^**^	−0.20
Employment	0.14	0.26^*^	−0.22	0.26^*^	0.22	0.05	−0.12	−0.01
Income	0.01	−0.36^**^	0.33^*^	−0.55^**^	−0.43^**^	−0.32^*^	0.45^**^	0.45^**^
Pain duration	−0.00	−0.26^*^	0.28^*^	−0.13	−0.35^**^	−0.14	0.07	0.16

* *p ≤ 0.05*,

** *p ≤ 0.01*.

*Sex coded: 0 = male, 1 = female; Race coded: 0 = white, 1 = black/other; Marital Status coded: 0 = married/partnered, 1 = not married/partnered; Employment coded: 0 = employed, 1 = not employed. BPS, Back Performance Scale; PROMIS, Patient-Reported Outcomes Measurement Information System; RMDQ, Roland-Morris Disability Questionnaire; BRS, Brief Resilience Scale; WHOQOL, World Health Organization Quality of Life questionnaire*.

The authors apologize for this error and state that this does not change the scientific conclusions of the article in any way. The original article has been updated.

