# Supplementary material for: Multisystem Resiliency as a Predictor of Physical and Psychological Functioning in Older Adults With Chronic Low Back Pain
Source: Front Psychol. 2019 Aug 22;10:1932. doi: 10.3389/fpsyg.2019.01932 (PMC6714590; doi:10.3389/fpsyg.2019.01932)
Supplement: Supplementary file 1 [file Table_1.DOCX]

**Supplementary Table 1.** Regression weights and means for resilience domains and individual measures across cluster groups

|  |  | |  | **Cluster 1** | |  | **Cluster 2** | |  | **Cluster 3** | |  | **Cluster 4** | |  |  |  |  |
| --- | --- | --- | --- | --- | --- | --- | --- | --- | --- | --- | --- | --- | --- | --- | --- | --- | --- | --- |
|  | Total  Sample  (n=60) | |  | High Resilience  (n=25) | |  | High Health Low PsySoc (n=13) | |  | High PsySoc Low Health (n=15) | |  | Low Resilience  (n=7) | |  |  |  |  |
|  | M | SD |  | M | SD |  | M | SD |  | M | SD |  | M | SD |  | *F* | *p* | *η_p_*^2^ |
|  |  |  |  |  |  |  |  |  |  |  |  |  |  |  |  |  |  |  |
| Psychological Domain | ― | ― |  | 0.55^a^ | 0.66 |  | -0.74^b^ | 0.51 |  | 0.49^a^ | 0.49 |  | -1.64^c^ | 0.91 |  | 31.42 | <.001 | .63 |
| Health Domain | ― | ― |  | 0.41^a^ | 0.59 |  | 0.62^a^ | 0.85 |  | -0.79^b^ | 0.97 |  | -0.93^b^ | 0.84 |  | 13.23 | <.001 | .42 |
| Social Support Domain | ― | ― |  | 0.84^a^ | 0.31 |  | -0.57^b^ | 0.58 |  | -0.08^c^ | 0.70 |  | -1.77^d^ | 0.55 |  | 55.21 | <.001 | .75 |
|  |  |  |  |  |  |  |  |  |  |  |  |  |  |  |  |  |  |  |
| **Psychological Domain** |  |  |  |  |  |  |  |  |  |  |  |  |  |  |  |  |  |  |
| Positive Affect | 35.68 | 7.81 |  | 38.36^a^ | 6.12 |  | 31.54^b^ | 4.01 |  | 39.73^a^ | 6.10 |  | 25.14^c^ | 9.72 |  | 12.21 | <.001 | .40 |
| Dispositional Hope | 49.67 | 10.44 |  | 54.44^a^ | 7.39 |  | 41.69^b^ | 9.74 |  | 54.87^a^ | 5.11 |  | 36.29^b^ | 9.71 |  | 16.87 | <.001 | .48 |
| Positive Well-Being | 87.75 | 18.99 |  | 99.32^a^ | 11.52 |  | 74.92^b^ | 12.45 |  | 94.47^a^ | 9.55 |  | 55.86^c^ | 16.76 |  | 30.97 | <.001 | .62 |
| Optimism | 17.30 | 3.64 |  | 18.96^a^ | 3.05 |  | 15.31^b^ | 3.07 |  | 18.40^a^ | 2.97 |  | 12.71^b^ | 2.56 |  | 10.70 | <.001 | .36 |
|  |  |  |  |  |  |  |  |  |  |  |  |  |  |  |  |  |  |  |
| **Health Domain** |  |  |  |  |  |  |  |  |  |  |  |  |  |  |  |  |  |  |
| Waist-Hip Ratio | 0.92 | 0.09 |  | 0.89^a^ | 0.07 |  | 0.87^a^ | 0.06 |  | 0.98^b^ | 0.10 |  | 0.97^b^ | 0.11 |  | 6.42 | <.01 | .26 |
| Body Mass Index | 29.32 | 5.83 |  | 27.62^a^ | 4.35 |  | 26.67^a^ | 5.99 |  | 32.88^b^ | 6.36 |  | 32.69^b^ | 4.55 |  | 5.13 | <.01 | .22 |
| Health Comorbidities | 1.02 | 0.95 |  | 0.72^a^ | 0.74 |  | 0.69^a^ | 0.75 |  | 1.40^b^ | 1.06 |  | 1.86^b^ | 1.07 |  | 4.73 | <.01 | .20 |
|  |  |  |  |  |  |  |  |  |  |  |  |  |  |  |  |  |  |  |
| **Social Support Domain** |  |  |  |  |  |  |  |  |  |  |  |  |  |  |  |  |  |  |
| Emotional Support | 31.02 | 8.29 |  | 37.76^a^ | 2.59 |  | 26.92^b^ | 5.45 |  | 30.00^b^ | 6.56 |  | 16.71^c^ | 4.68 |  | 41.81 | <.001 | .69 |
| Instrumental Support | 14.52 | 5.35 |  | 18.28^a^ | 2.83 |  | 11.23^b*^ | 5.21 |  | 14.07^c^ | 4.68 |  | 8.14^b^ | 3.67 |  | 16.09 | <.001 | .46 |
| Informational Support | 15.45 | 4.20 |  | 18.72^a^ | 1.51 |  | 13.46^b^ | 2.57 |  | 15.40^c^ | 3.02 |  | 7.57^d^ | 2.07 |  | 48.89 | <.001 | .72 |
|  |  |  |  |  |  |  |  |  |  |  |  |  |  |  |  |  |  |  |
|  |  |  |  |  |  |  |  |  |  |  |  |  |  |  |  |  |  |  |

*Note:* Means in the same row that do not share a superscript are significantly different at *p*<.05. *comparison with Cluster 3 is different at *p*=.06.

**Supplementary Table 2.** Descriptive statistics for pain, function, and psychological measures across cluster groups

|  |  | **Cluster 1** | |  | **Cluster 2** | |  | **Cluster 3** | |  | **Cluster 4** | |  |  |  |  |
| --- | --- | --- | --- | --- | --- | --- | --- | --- | --- | --- | --- | --- | --- | --- | --- | --- |
|  |  | High Resilience  (n=25) | |  | High Health Low PsySoc (n=13) | |  | High PsySoc Low Health (n=15) | |  | Low Resilience  (n=7) | |  |  |  |  |
|  |  | M | SD |  | M | SD |  | M | SD |  | M | SD |  | *F* | *p* | *η_p_*^2^ |
|  |  |  |  |  |  |  |  |  |  |  |  |  |  |  |  |  |
| **Pain and Function** |  |  |  |  |  |  |  |  |  |  |  |  |  |  |  |  |
| BPS Functional Performance |  | 2.32^a^ | 1.98 |  | 3.42^a^ | 2.87 |  | 4.79 | 2.92 |  | 6.79^b^ | 2.53 |  | 5.08 | .004 | .25 |
| BPS ME-Pain |  | 19.18 | 17.54 |  | 33.59 | 25.81 |  | 34.88 | 27.63 |  | 43.93 | 21.33 |  | 2.43 | .077 | .14 |
| PROMIS Physical Function |  | 15.24 | 3.03 |  | 15.31^†^ | 3.98 |  | 13.36 | 3.33 |  | 11.05^†^ | 3.92 |  | 2.99 | .041 | .17 |
| PROMIS Pain Intensity |  | 8.11 | 2.06 |  | 9.09 | 2.65 |  | 9.35 | 1.79 |  | 8.89 | 1.60 |  | 1.17 | .332 | .07 |
| RMDQ Disability |  | 7.27^a^ | 4.67 |  | 7.99^a^ | 5.33 |  | 10.06 | 4.05 |  | 13.80^b^ | 5.23 |  | 3.86 | .015 | .21 |
|  |  |  |  |  |  |  |  |  |  |  |  |  |  |  |  |  |
| **Psychological** |  |  |  |  |  |  |  |  |  |  |  |  |  |  |  |  |
| PROMIS Depression |  | 9.50^a^ | 1.69 |  | 14.40^b^ | 5.29 |  | 13.88^a^ | 4.03 |  | 22.93^c^ | 8.78 |  | 12.10 | <.001 | .45 |
| BRS Resilience |  | 4.04^a^ | 0.58 |  | 3.28^b^ | 0.66 |  | 4.11^a^ | 0.61 |  | 3.17^b*^ | 0.99 |  | 4.67 | .006 | .24 |
| WHOQOL Quality of Life |  | 4.44^a^ | 0.98 |  | 3.81^a^ | 0.69 |  | 4.24^a^ | 0.58 |  | 2.75^b^ | 1.21 |  | 6.27 | .001 | .30 |
|  |  |  |  |  |  |  |  |  |  |  |  |  |  |  |  |  |
|  |  |  |  |  |  |  |  |  |  |  |  |  |  |  |  |  |

*Note:* Means in the same row that do not share a superscript are significantly different at *p*<.05, ^†^*p*=.07, *comparison with Cluster 1 is different at *p*=.07. BPS=Back Performance Scale; ME=Movement-Evoked; PROMIS=Patient-Reported Outcomes Measurement Information System; RMDQ=Roland-Morris Disability Questionnaire; BRS=Brief Resilience Scale; WHOQOL=World Health Organization Quality of Life questionnaire.
